# Paralympic sport in Central Asia: a scoping review with descriptive analysis of participation, achievement, and medal efficiency (1996–2024)

**DOI:** 10.3389/fspor.2026.1893368

**Published:** 2026-09-22

**Authors:** Ulbossyn Marchibayeva

**Affiliations:** Department of Physical Education and Sport, L.N. Gumilyov Eurasian National University, Astana, Kazakhstan

**Keywords:** Central Asia, disability sport, elite sport development, Paralympic sport, participation inequalities, PRISMA-ScR, sport policy

## Abstract

Published research on Paralympic sport in Central Asia remains limited despite the region's growing presence at the Games. This review focuses on elite, Games-level participation and achievement. We conducted a scoping review reported in line with the PRISMA extension for scoping reviews, alongside an analysis of International Paralympic Committee (IPC) archival data for five Central Asian countries (Kazakhstan, Uzbekistan, Kyrgyzstan, Tajikistan, Turkmenistan) across Summer (1996–2024) and Winter (1994–2026) Games, framed through the SPLISS model and the constraint-led approach. The scoping review maps international knowledge; the IPC analysis documents regional outcomes, offering complementary evidence. The review identified three domains: income-, gender-, and technology-linked participation gaps (D1); discipline-specific age profiles ranging from a median of 25 years in Para Swimming to 38 years in Para Equestrian (D2); and ten national policy factors linked to Paralympic success (D3). IPC data showed Central Asian participation rising from 15 athletes in 1996 to 113 in 2024, led by Uzbekistan (*n* = 64, Paris 2024) and Kazakhstan (*n* = 44), concentrated in Para Athletics, Para Judo, and Para Powerlifting, plus a full Kazakhstani sitting volleyball squad and Para Swimming. Female representation rose from 0% in 1996 to 41.6% in 2024, near the ∼43% global benchmark. Medal efficiency was 0% before 2012, spiked to 73.5% at Rio 2016 (95% CI 59.7%–83.8%), driven by Uzbekistan winning more medals than athletes that edition, then settled at 32.9% (Tokyo 2020) and 31.0% (Paris 2024, 95% CI 23.2%–40.0%); the 2016 spike is statistically distinguishable from other editions, while Tokyo 2020 and Paris 2024 are not. Uzbekistan grew from 0 athletes in 1996 to 64 in 2024, with the sharpest rise from 32 (Rio 2016) to 64 (Paris 2024), coinciding with documented 2019–2024 state Paralympic-development measures-a plausible institutional account alongside classification reform and host-proximity effects. No study identified by our search has examined elite Paralympic sport development in Central Asia specifically. Four research priorities are proposed: epidemiological profiling, adapted policy analysis with regional researchers, longitudinal athlete tracking, and psychological support programs.

## 1 Introduction

The Paralympic Games grew from 400 athletes representing 23 countries in Rome 1960 to 4,400 athletes from 170 nations at Paris 2024 (6–8). Article 30 of the UN Convention on the Rights of Persons with Disabilities guarantees the right to sport and recreation (9), and Paralympic performance serves as an indicator of how states treat citizens with disabilities (10). This article focuses specifically on elite, Games-level participation and achievement—the pathway from national team selection to the Paralympic Games—rather than on grassroots participation, school-level adapted physical education, or attrition from recreational sport, which fall outside the scope of the available IPC data and are addressed only indirectly through the scoping review evidence.

Published research on Paralympic sport is concentrated in a few high-income nations—Australia, the United Kingdom, Canada, and Western Europe (11–13). Central Asia, five post-Soviet states with a combined population above 75 million, has received almost no attention in the international literature. The region shares a post-Soviet approach to disability marked by medical-model thinking, institutional segregation, and limited civic space (14). Within Kazakhstan, Marchibayeva (15) documented a shortage of qualified specialists, minimal research output, and few adapted facilities in the educational system—factors that limit the pathway from grassroots participation to elite competition.

Recent work has addressed specific aspects. Oggero et al. (16) reported income-, gender-, and technology-linked inequalities in Summer Games participation (1960–2016). Kons et al. (7) conducted the largest analysis of age distributions across 13 disciplines at Paris 2024. Pankowiak et al. (12) identified ten national policy interventions linked to Paralympic success. Krasmik et al. (17) examined motivation and hardiness among Kazakhstani Para athletes. Revuelta and Hernández (18) compared Paralympic sport promotion policy across ten Latin American countries and found that even where the right to sport is formally recognized, talent-identification and school-to-national-team pathways remain the weakest links—a pattern this article revisits for Central Asia in Section 4.4. None of these studies, however, examined how their findings apply to Central Asia.

This review is intended primarily for national Paralympic committees, sport ministries, and researchers in the five countries studied, as well as for international bodies (IPC, regional federations) designing capacity-building programs; the evidence map and IPC analysis are structured so that findings can be read at the level of individual countries where the underlying data allow it.

### 1.1 Theoretical framing

We used two models as an analytical lens, chosen for complementary reasons. First, the SPLISS model (4), adapted for the Paralympic context by Patatas et al. (11) and Pankowiak et al. (12), provided structure for evaluating national policy conditions; it was selected because it is the most widely used comparative framework for elite sport policy and allows Central Asian conditions to be benchmarked, pillar by pillar, against countries studied elsewhere. Its pillars—financial support, governance, participation, talent identification, coaching, facilities, competition, and research—served as a checklist against which we compared the Central Asian data; this review draws primarily on the participation, talent identification, coaching, facilities, and research pillars, for which archival and published evidence exists, and treats governance, competition opportunities, and organization as pillars requiring dedicated future data collection (Section 4.6). Second, the constraint-led approach (5), which treats impairment type, classification, funding, and athlete pool size as interacting constraints, guided our interpretation of participation patterns and age profiles; it was selected because, unlike SPLISS, it operates at the level of individual athlete development and is therefore suited to interpreting discipline concentration and age-profile findings that a policy-level framework alone cannot explain. Neither model was designed with post-Soviet transition economies in mind, and Section 4.6 returns to the limits of that fit.

The study had two aims: (1) to map evidence across three domains—participation and achievement, age and athlete development, and national sport policy; and (2) to analyze IPC data for five Central Asian countries across Summer and Winter Games, adding medal efficiency, with uncertainty estimates, as a new metric.

## 2 Materials and methods

### 2.1 Study design

We adopted the scoping review of Arksey and O’Malley (1), enhanced by Levac et al. (2), and supplemented it with a descriptive analysis of IPC data (19). Reporting followed PRISMA-ScR (3). The checklist is available as Supplementary Material. No review protocol was registered prospectively. Figure 1 shows the study selection process.

**Figure 1:**
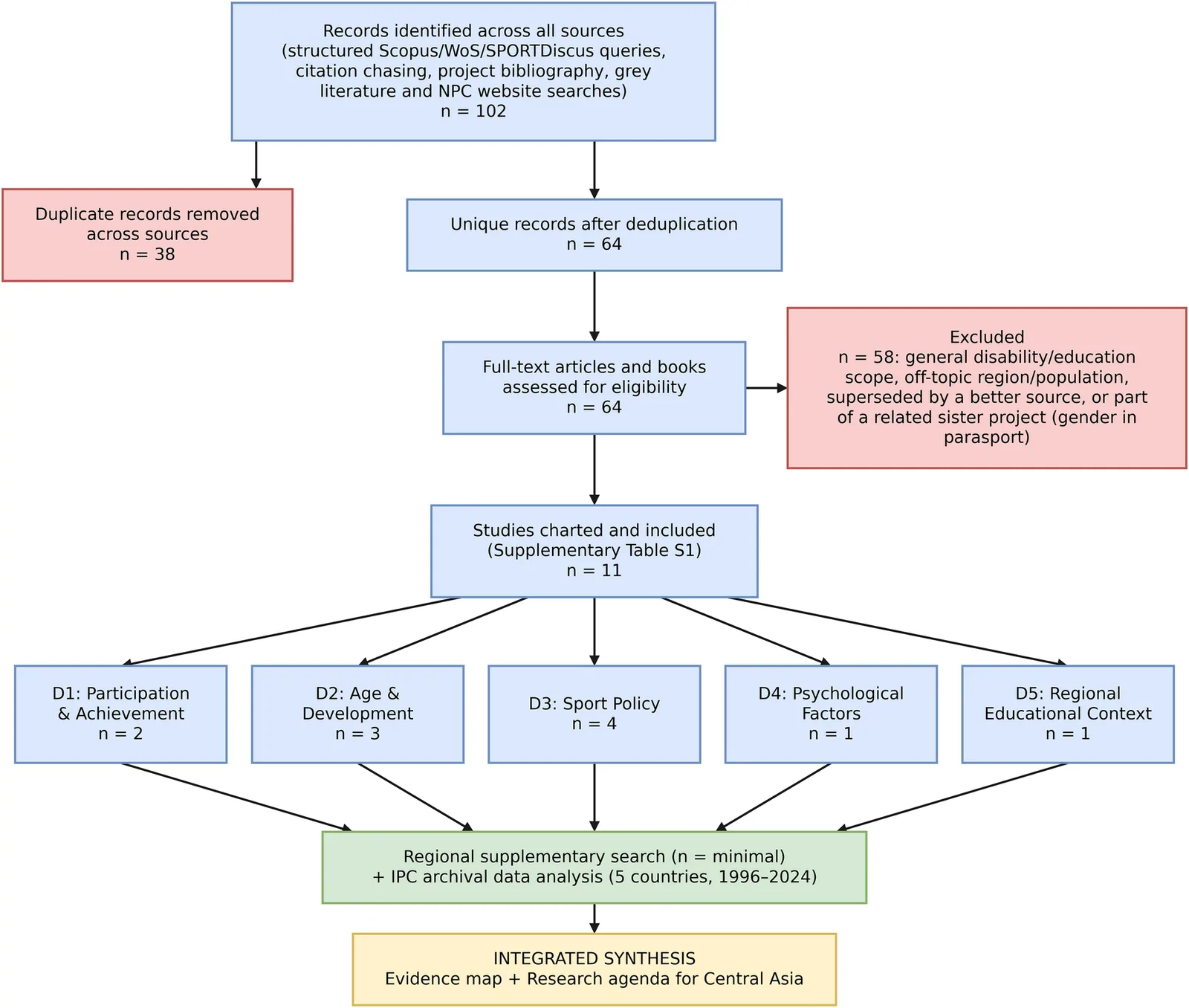
PRISMA-ScR flow diagram for the scoping review, reported as two identification routes. Route 1 (systematic search) identified records across multiple sources: structured Scopus, Web of Science Core Collection, and SPORTDiscus queries; backward and forward citation chasing; a pre-existing project bibliography; and targeted grey-literature and national Paralympic committee website searches. Route 2 comprised five sources identified outside the systematic search: four policy and cross-regional comparators added during peer review, and the author's prior work on the regional educational context (Section 2.5). D1, participation and achievement; D2, age and development; D3, sport policy; D4, psychological factors; D5, regional educational context; IPC, International Paralympic Committee.

Two data sources were used deliberately, not in parallel. The scoping review (Section 3.1) answers the question “what is known internationally about Paralympic participation, development, and policy?”, establishing the comparative and theoretical context. The IPC archival analysis (Section 3.2) answers the complementary but distinct question “what has actually happened in Central Asia?”, a question the published literature cannot answer because, as the review itself demonstrates, almost no empirical study has been conducted in the region. The Results and Discussion sections are structured to read the two sources together: the review supplies the interpretive lens (SPLISS pillars, constraint-led mechanisms, comparator findings from other regions) that the Discussion then applies to the IPC findings, rather than presenting the two as independent, unconnected analyses.

### 2.2 Research questions

RQ1: What is the extent and distribution of published research on Paralympic participation and achievement at the elite level, with particular attention to Central Asia and other middle-income, post-Soviet countries where comparable evidence might exist?

RQ2: What is known about age-related performance patterns in Para athletes internationally, and how can these data inform the design of emerging Paralympic systems such as those in Central Asia?

RQ3: What national policy models have been linked to Paralympic success internationally, and to what degree do they account for—or need adaptation to—the post-Soviet Central Asian context?

All three questions establish the international evidence base first and then treat Central Asia as the lens through which that evidence is interpreted. RQ2 and RQ3 draw on international literature because no Central Asia-specific studies exist on these two questions—a finding in itself, reported in Section 4.1. RQ1 is the only question answered with region-specific primary data, via the IPC analysis in Section 3.2.

### 2.3 Search strategy

We conducted a scoping review following the methodological framework of Arksey and O’Malley, as enhanced by Levac et al., and reported in line with the PRISMA-ScR extension for scoping reviews. Our search combined structured database queries in Scopus, Web of Science Core Collection, and SPORTDiscus (2010–2026) with backward and forward citation chasing, screening of a pre-existing project bibliography, and targeted searches of grey literature (UN/IPC reports) and the national Paralympic committee websites of Kazakhstan, Uzbekistan, and Kyrgyzstan (20, 21). Search terms were organized into two blocks, combined with the Boolean operator AND. Block 1 covered population terms describing the athlete group of interest: Paralympic, parasport, disability sport, adapted sport. Block 2 covered the phenomena of interest: participation, achievement, medal, peak performance, age, policy, talent identification, development. A supplementary search added Central Asian country names and the terms Central Asia and post-Soviet.

### 2.4 Eligibility criteria

We included: (a) peer-reviewed empirical studies, reviews in English or Russian; (b) focus on at least one domain; (c) Scopus or WoS indexing; (d) 2010–2026. For the regional search, all study types were eligible regardless of quartile. We excluded: (a) conference abstracts; (b) editorials; (c) rehabilitation-only studies; (d) Special Olympics or Deaflympics studies only.

### 2.5 Selection of sources of evidence

Screening and selection of sources of evidence were conducted by the sole author (UM). No second reviewer participated at any stage, and screening was therefore not performed independently in duplicate. This is acknowledged as a limitation in Section 4.8.

Records were identified through two routes, which are reported separately in Figure 1. The first route comprised the systematic search described in Section 2.3, which yielded 102 records across three information sources: 36 full texts held in the project archive, 36 entries from a disability-and-inclusion reference catalogue, and 30 entries from a topic-organized bibliography assembled through citation chasing and targeted grey-literature and national Paralympic committee website searches. Deduplication by normalised DOI, with one manual title-based merge for a record lacking a DOI in one source, removed 38 records appearing in more than one source and left 64 unique records.

Because the identification strategy relied substantially on a pre-existing project archive and on citation chasing, the full document was available for the great majority of these 64 records. Eligibility was therefore assessed on the full document, and no separate title/abstract triage stage with independent record counts was conducted; where topical relevance was ambiguous at first inspection, the record was carried through to full assessment rather than set aside, so that uncertainty was consistently resolved in favour of retention. Of the 64 unique records, 6 met the eligibility criteria in Section 2.4 and 58 were excluded, most commonly because the study addressed disability inclusion, education or rehabilitation outside elite Games-level sport, or fell outside all three review domains. A record-level list of all 102 identified records, the deduplication mapping and the include/exclude status of each of the 64 unique records is provided as Supplementary Material S2.

The second route comprised five further sources identified outside the systematic search: four (18, 22–24) were added during peer review in response to reviewer requests for additional policy and cross-regional comparators, and one (15) is the author's own prior work on the regional educational context. Each was assessed against the same eligibility criteria in full text before inclusion. Eleven studies in total were therefore retained for data charting and synthesis: 6 from the systematic search and 5 from other methods.

Because a single reviewer conducted the entire selection process, no between-reviewer discrepancies arose, and consequently no consensus or arbitration procedure was applicable. A generative AI tool (Perplexity) was used during record identification and to assist in summarising candidate records; its role is described in the Generative AI disclosure. Final eligibility decisions were made by the author on the basis of the full document.

### 2.6 Data charting process

Data were charted by the sole author (UM) using a charting form developed for this review before extraction began. Charting was not performed in duplicate, and no second person independently extracted or verified the charted values; as with screening, this follows from the single-author design. Because a single charter was involved, no inter-charter discrepancies arose and no reconciliation procedure was required. The following variables were charted for each included study: author(s), publication year, journal, journal quartile, geographic and institutional context, study design, sample, review domain, key findings, MMAT quality rating, and relevance to the Central Asian context. Values were recorded as reported in the source; where a value could not be reconciled with a charting field it was left blank rather than inferred, and study authors were not contacted. The form was reviewed against the first studies charted and its field structure confirmed before the remaining studies were processed, in the iterative sense described by Levac et al. (2). Methodological quality was appraised with the MMAT (25), likewise by the sole author and without duplicate appraisal; consistent with established scoping review practice (1, 3), appraisal characterises the evidence base rather than determining inclusion, and no study was excluded on quality grounds. The completed charting table for all eleven included studies is provided as Supplementary Table S1.

### 2.7 IPC data analysis

We analyzed data from the IPC Historical Results Archive (HIRA) for five countries across eight Summer Games (1996–2024) and nine Winter Games (1994–2026). Variables: athletes per country per Games, sex, disciplines, medals by type. Medal efficiency was computed as total medals divided by total athletes per edition, following Oggero et al. (16). Because both medal counts and female-athlete counts are small in several editions, we computed 95% confidence intervals for these proportions using the Wilson score method, which is more stable than the normal approximation at small n. For the female-share estimates this is a standard binomial proportion (each athlete is either female or not) and the Wilson interval applies directly. For medal efficiency, one caveat applies: because a single athlete can win more than one medal, the medal count is not strictly bounded by the athlete count in the way a true binomial numerator is, even though it never exceeds it in the present dataset; the Wilson interval for medal efficiency is therefore an approximate uncertainty band, not a formally correct binomial confidence interval. Both sets of intervals are reported alongside point estimates throughout Section 3.2 and shown as shaded bands in Figures 2, 3, and should be read as a description of sampling uncertainty in a descriptive dataset rather than as a test of a causal hypothesis. To test formally whether female representation and medal efficiency changed across editions rather than fluctuating randomly, we additionally fitted a logistic regression of each proportion on edition order (equivalent to a Cochran-Armitage trend test), using the same eight editions as trials, and checked each model for overdispersion using the Pearson *χ*^2^-to-degrees-of-freedom ratio. For female representation, a genuine binomial proportion, overdispersion was mild (*χ*^2^/df = 1.5); we report the corresponding overdispersion-adjusted standard error, which is the more conservative and appropriate choice. For medal efficiency, overdispersion was severe (*χ*^2^/df = 11.3), which is expected given the multiple-medals-per-athlete caveat above and confirms quantitatively, not just qualitatively, that a binomial trend test is not formally valid here; we report the same model as a descriptive trend indicator only. Analysis used frequencies, proportions, these interval estimates, and the trend models in Python (SciPy, statsmodels) and Microsoft Excel. The IPC itself notes that HIRA records from 2014 onward are verified as accurate, while statistics for earlier editions (1996–2012) remain under institutional review (26); this affects mainly the earliest, smallest delegations and is noted where relevant in Section 4.8. No ethical approval was needed as data are secondary and publicly archived.

**Figure 2:**
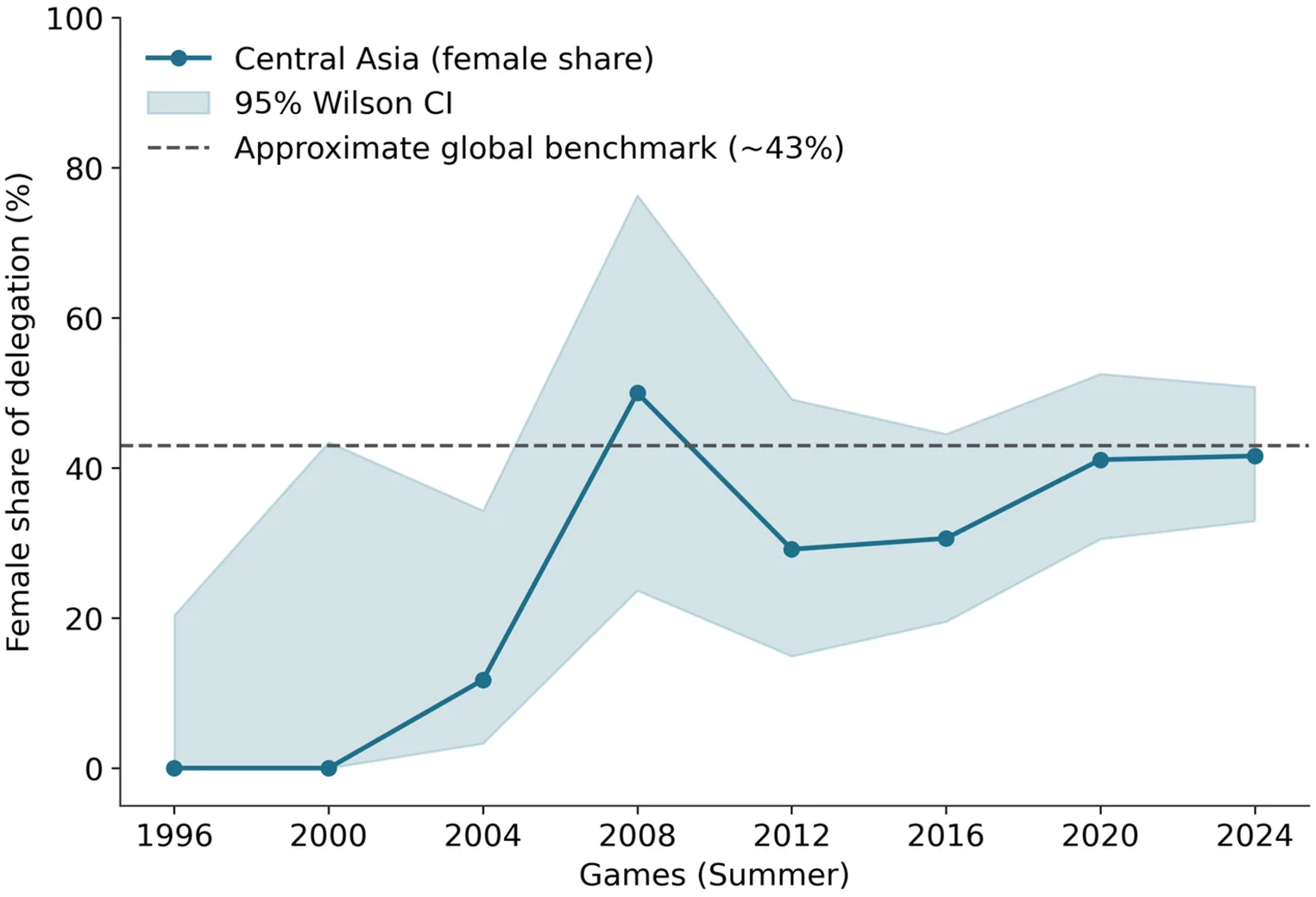
Female representation among Central Asian Paralympians versus the approximate global benchmark, 1996–2024, with 95% Wilson confidence intervals (shaded band) for the Central Asian estimate. Source: IPC Historical Results Archive; global benchmark approximated from Oggero et al. (16) and Kons et al. (7).

**Figure 3:**
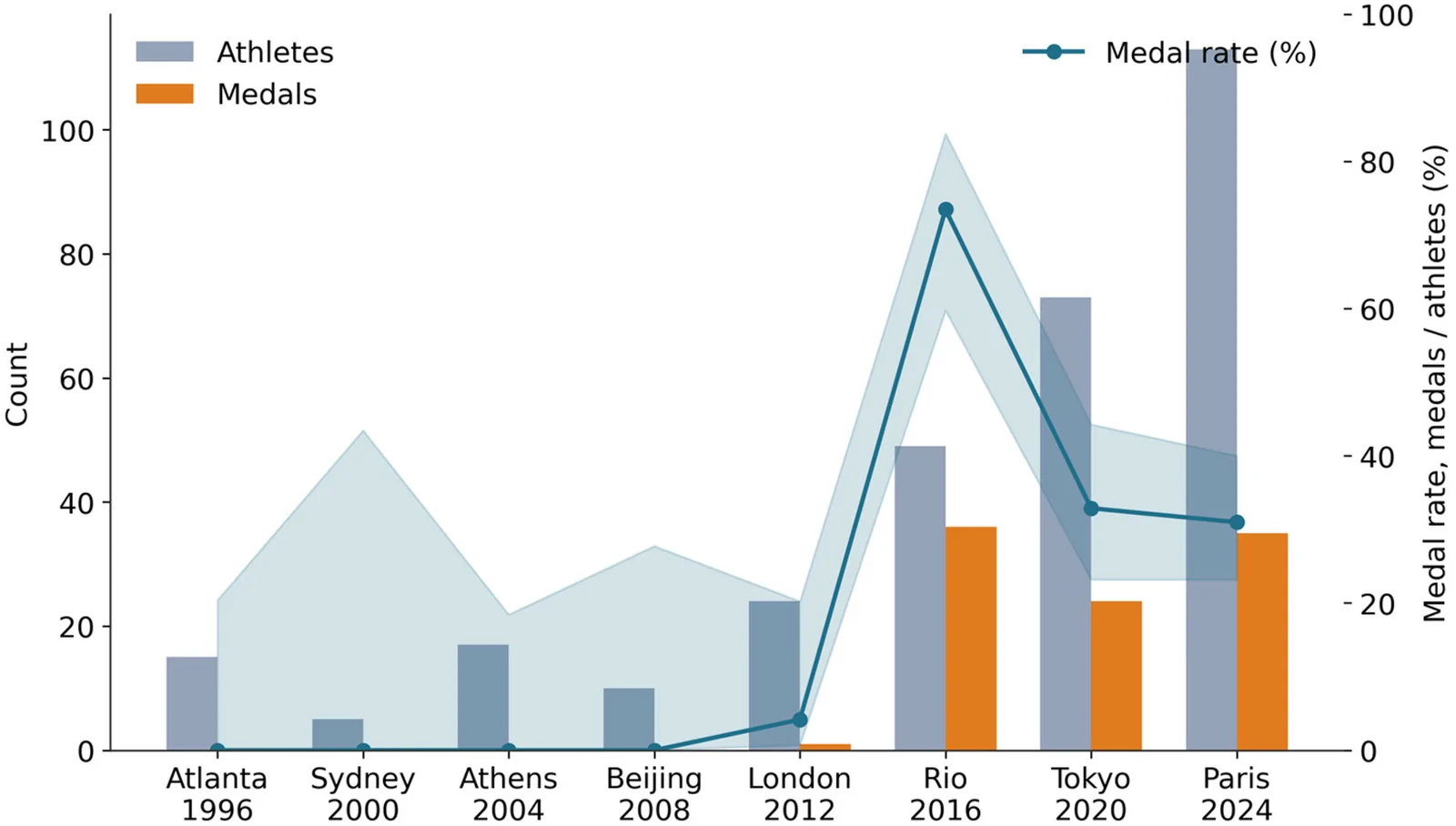
Athletes, medals, and medal rate (%) for Central Asian countries at Summer Paralympic Games (1996–2024), with approximate 95% Wilson confidence intervals (shaded band; heuristic uncertainty bands—see Section 2.7) for medal rate. Source: IPC Historical Results Archive.

## 3 Results

### 3.1 Scoping review evidence

#### 3.1.1 Participation and achievement (Domain 1)

Across all information sources combined, the search identified a total of 102 records before deduplication and screening (Figure 1): 36 full texts retained in the project's archive, 36 entries from a broader disability-and-inclusion reference catalogue, and 30 entries from a topic-organized bibliography assembled through citation chasing and targeted grey-literature and NPC-website searches. After removing 38 records duplicated across these three sources, 64 unique records were assessed for eligibility in full text, of which 6 met the inclusion criteria. A further 5 studies were identified outside the systematic search—four added during peer review as policy and cross-regional comparators, and one the author's prior work on the regional educational context—giving 11 studies charted in total (Section 2.5; Figure 1; Supplementary Material S2). Oggero et al. (16) analyzed Summer Games 1960–2016 data and reported significant differences in participation by income (*p* = 0.000). Women made up ∼29% of all Paralympians. In events requiring assistive products, high-income countries had higher participation (*p* = 0.000) and achievement (*p* = 0.007). Dehghansai et al. (6) synthesized 21 studies and found a disproportionate focus on short-term interventions.

#### 3.1.2 Age and athlete development (Domain 2)

Kons et al. (7) analyzed 5,540 athletes across 13 disciplines at Paris 2024. Age varied by discipline [*χ*^2^(12) = 1,027.035, *p* < 0.001]: Para Equestrian Mdn = 38, Para Archery 37, Para Powerlifting 36, versus Para Swimming 25, Para Taekwondo 27, Para Athletics 29. Males were slightly older (Mdn = 30 vs. 28; *r* = 0.08, trivial) (27). No age difference separated medallists from non-medallists (*p* = 0.289). Dehghansai et al. (28, 29) documented the complexity of Paralympic talent identification (30). Patatas et al. (22) showed that Global North models cannot be transferred directly.

#### 3.1.3 National sport policy (Domain 3)

Pankowiak et al. (12) interviewed 23 managers from four leading Paralympic nations and identified ten policy factors. Two were unique to the Paralympic domain: disability-knowledge integration and national classification capacity. Thomas and Guett (23) noted that disability sport policy research lags behind able-bodied sport. Hammond and Jeanes (24) showed that performance-based funding can exclude non-elite athletes. Revuelta and Hernández (18) found that even where ten Latin American countries had, on paper, formally recognized the right to sport for persons with disabilities, talent-identification systems and the school-club-national-team pathway remained the least developed part of their policy frameworks—a comparator finding revisited in Section 4.4. No policy study has targeted any Central Asian country specifically.

#### 3.1.4 Psychological factors

Krasmik et al. (17) examined 60 Kazakhstani Para athletes. Self-improvement at optimal intensity was the leading motive. Hardiness and control correlated positively with motivation, but overall hardiness stayed low across qualification levels. Paralympic federations in Kazakhstan typically lack permanent sport psychologists.

#### 3.1.5 Regional educational context

Marchibayeva (15) analyzed Kazakhstani legislation and educational practice and identified four barriers to adaptive physical culture: shortage of qualified specialists, minimal scientific research, underdeveloped teaching materials, and insufficient adapted facilities. These barriers limit athlete progression into elite Paralympic sport and align with the grassroots-participation and coaching pillars of the SPLISS model.

### 3.2 IPC archival data: participation and achievement

#### 3.2.1 Participation trends

Total Central Asian participation increased from 15 athletes at Atlanta 1996 to 113 at Paris 2024 (Table 1; Figure 4). Uzbekistan grew from zero to 64 athletes; Kazakhstan from 13 to 44, though not monotonically—Kazakhstan's own delegation fluctuated sharply in the earlier editions (13 in 1996, falling to 2 in 2000, rising to 8 in 2004, falling again to 3 in 2008, before the sustained rise from 2012 onward), a pattern of similar magnitude to the Winter Games volatility discussed in Section 3.2.5 and likely reflecting the same mix of small-delegation noise, qualification-cycle effects, and pre-2014 IPC data that remains under institutional review (Section 2.7), rather than a specific programmatic explanation we can identify from the available data. Kyrgyzstan (1–4) and Turkmenistan (0–5) kept minimal delegations throughout, and Tajikistan (0–1) last competed at Tokyo 2020. Two acceleration phases occurred: 2008–2016 (10 to 49, +390%) and 2016–2024 (49 to 113, +131%). Both phases overlap candidate confounds that are examined explicitly in Section 4.2 and Table 2: the transition to the 2015 IPC Athlete Classification Code and the 2018 World Para Athletics classification revision, and—for the 2024 edition specifically—the geographic proximity of the Paris Games to Central Asia relative to Rio de Janeiro or Tokyo.

**Table 1:** Central Asian participation and medal efficiency in Summer Paralympic Games (1996–2024), with approximate 95% Wilson confidence intervals for medal efficiency (M/A %; heuristic uncertainty bands—see Section 2.7).

Games	KAZ	UZB	KGZ	TJK	TKM	Total	F %	Medals	M/A % (95% CI)
1996	13	—	2	—	—	15	0.0	0	0.0 (0.0–20.4)
2000	2	—	2	—	1	5	0.0	0	0.0 (0.0–43.4)
2004	8	1	3	1	4	17	11.8	0	0.0 (0.0–18.4)
2008	3	2	1	1	3	10	50.0	0	0.0 (0.0–27.8)
2012	7	10	1	1	5	24	29.2	1	4.2 (0.7–20.2)
2016	11	32	3	1	2	49	30.6	36	73.5 (59.7–83.8)
2020	26	44	2	1	0	73	41.1	24	32.9 (23.2–44.3)
2024	44	64	4	—	1	113	41.6	35	31.0 (23.2–40.0)

All athlete, gender, and medal counts, including the F % column, are based on the IPC HIRA country-level participant tables (men/women breakdown) for all five countries; Kyrgyzstan, Tajikistan, and Turkmenistan have not won a Paralympic medal to date. KAZ, Kazakhstan; UZB, Uzbekistan; KGZ, Kyrgyzstan; TJK, Tajikistan; TKM, Turkmenistan; —, non-participation. Source: IPC Historical Results Archive (https://www.ipc-services.org/hira/countries).

**Figure 4:**
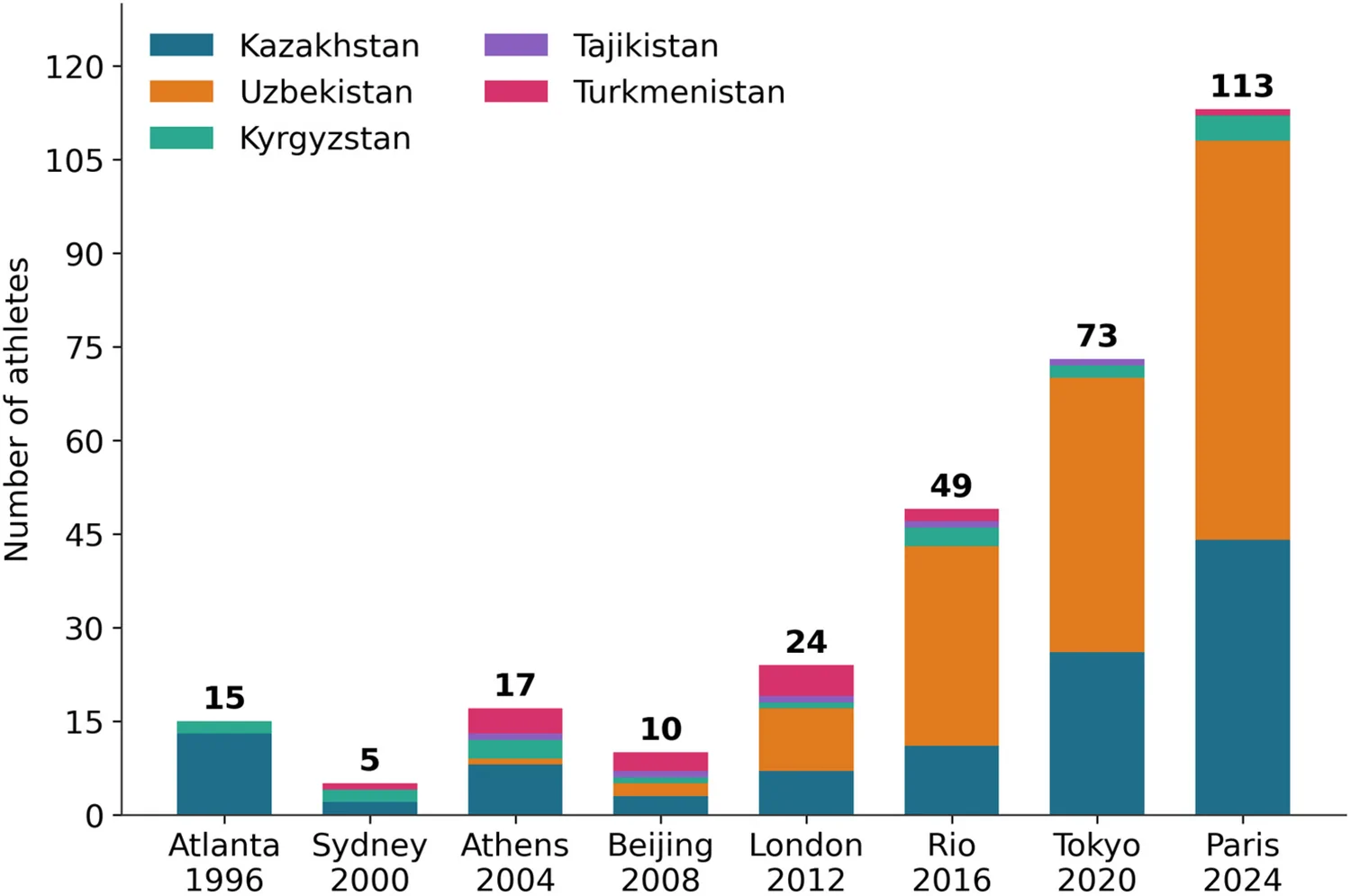
Participation of Central Asian countries in Summer Paralympic Games (1996–2024). Numbers above bars show total regional delegation size. Source: IPC Historical Results Archive.

**Table 2:** IPC and World Para Athletics classification reform timeline, 1996–2025, relevant to the Central Asian participation trend in Table 1 and Figure 4.

Date	Reform
2007	First unified IPC Athlete Classification Code adopted.
2011	IPC Position Stand on evidence-based classification adopted, setting principles for future sport-specific reforms.
2013–2015	Code revision under Article 17.6 (feedback round opened 2013); IPC Athlete Classification Code 2015 adopted—in force through Paris 2024.
1 Jan 2018	World Para Athletics: new maximum-allowable-standing-height (MASH) formula for double-leg amputees; new T61–64 sport class group; several existing classes (T/F20, T/F31–34, T/F35–38) returned to review status.
From 1 Jan 2025	New IPC Athlete Classification Code enters into force, superseding the 2015 Code (post-dates the participation data analyzed here).

Sources: International Paralympic Committee (35–37).

#### 3.2.2 Gender composition and benchmarking

At Atlanta 1996 all athletes were male. Female participation first appeared at Athens 2004 (2/17, 11.8%, 95% CI 3.3%–34.3%) and reached 41.6% at Paris 2024 (47/113, 95% CI 32.9%–50.8%; Figures 2, 5; Section 3.2.1; Table 1).

**Figure 5:**
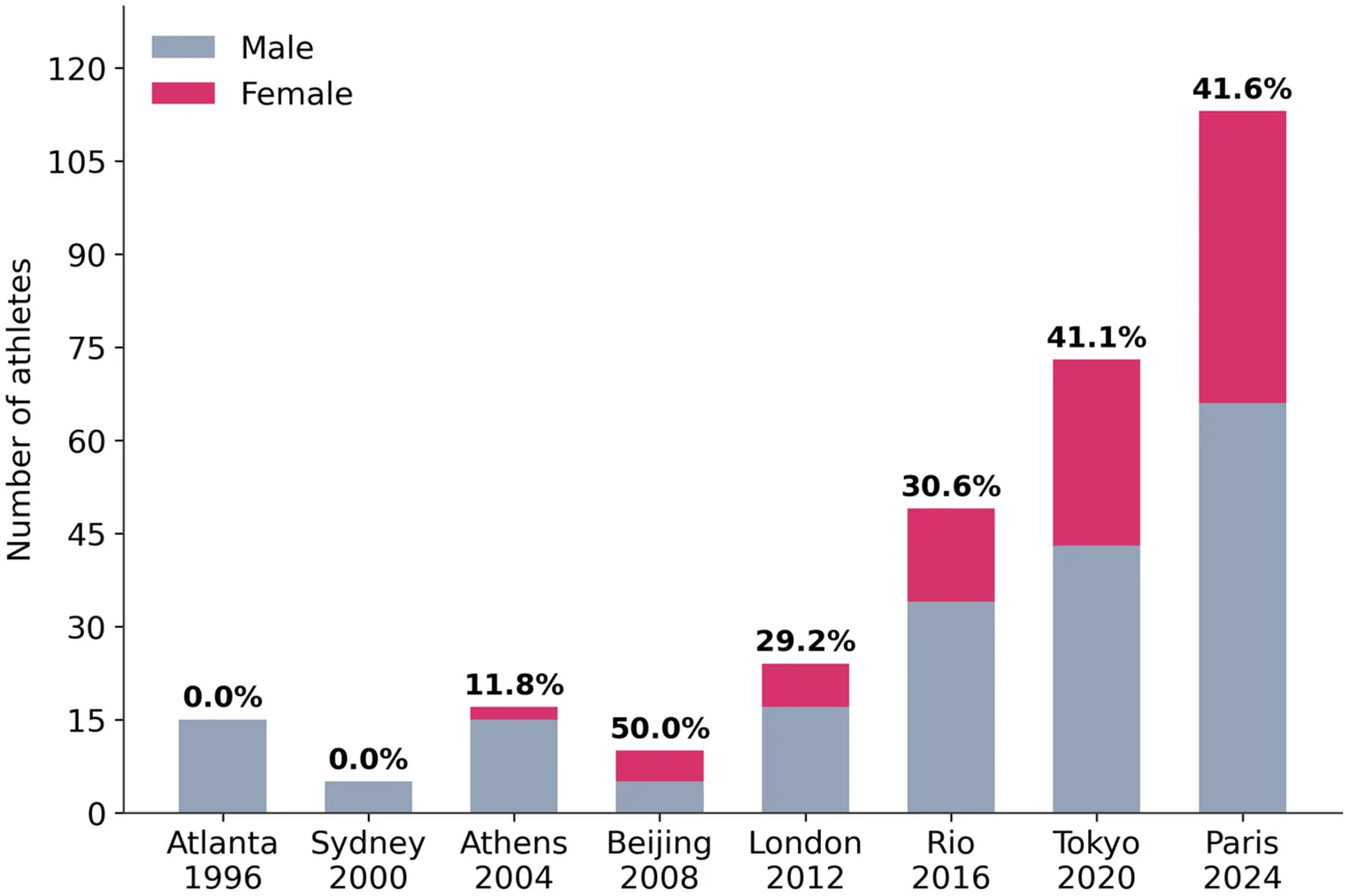
Gender composition of Central Asian Paralympic delegations, 1996–2024. Percentages above bars show female share for each edition (Table 1). Source: IPC Historical Results Archive.

Because several editions involved fewer than 15 athletes, the female-share estimates carry wide uncertainty: the apparent 2008 peak (50%) is based on *n* = 10 athletes and has a 95% CI of 23.7%–76.3%, meaning the true underlying share for that edition could plausibly have been anywhere from about one-quarter to three-quarters female. Compared with the approximate global benchmark of ∼43% (derived from Oggero et al. (16) and Kons et al. (7), and corroborated by Santos et al. (31), who reports 44.48% female participation at Paris 2024 across all National Paralympic Committees), the 2024 estimate (41.6%, 95% CI 32.9%–50.8%) overlaps the global figure closely (Figure 2). A logistic regression trend test across the eight editions (Section 2.7) indicates a statistically significant increase in female representation over time; because the data show mild overdispersion relative to a strict binomial model (Pearson *χ*^2^/df = 1.5), we report the overdispersion-adjusted estimate (*b* = 0.29 per edition, adjusted *z* = 3.04, *p* = 0.002) rather than the more optimistic unadjusted value, so the overall direction of change is not attributable to chance despite the non-monotonic, edition-to-edition pattern. Given the overlap with the global benchmark, no timeline to convergence is proposed; whether Central Asian female representation is converging, and on what timescale, cannot be determined from eight data points with this much edition-to-edition variability, even though the underlying upward trend itself is statistically supported. Discipline-level breakdown of female participation (e.g., Para Judo versus Para Swimming) was not available at the precision required for a reliable per-discipline, per-edition count and is noted as a direction for future data collection in Section 4.8. At least three distinct explanations could account for the gap between Central Asian and global female representation, and the archival data used here cannot distinguish among them: policy and cultural barriers to women's participation in sport more broadly; compositional effects, since the region's three core disciplines (Para Athletics, Para Judo, Para Powerlifting) have historically skewed male internationally; and the local epidemiology of disability itself, since war- and conflict-related impairments in the region are disproportionately male, which could shift the eligible athlete pool's sex ratio independently of any policy barrier. Distinguishing between these would require disability-prevalence data by sex and impairment type for each country, which were not available for this review. Santos et al. (31) reports that Asia recorded the strongest regional growth in female Paralympic participation between 1988 and 2024, while the number of National Paralympic Committees entering no female athletes rose overall, and concludes that region-specific gender-equity policy is required; the Central Asian trajectory reported here is consistent with that continental pattern.

#### 3.2.3 Discipline concentration

At Paris 2024, Central Asian athletes were concentrated in Para Athletics (30, 26%), Para Judo (19, 17%), and Para Powerlifting (15, 13%), together accounting for 56% of the regional delegation, with Para Sitting Volleyball (12, 10%—the full Kazakhstan men's team) and Para Swimming (11, 10%) next, followed by Para Taekwondo (9, 8%), Para Canoe (8, 7%), Para Shooting (4, 3%), Para Cycling Road (2, 2%), Para Cycling Track (2, 2%), and one athlete each (<1%) in Para Rowing, Para Table Tennis, and Para Triathlon (Figures 6, 7), based on IPC HIRA per-discipline, per-country participation tables for all 23 disciplines contested at Paris 2024 (https://www.ipc-services.org/hira/paralympics/participants/code/PG2024). The discipline-level total (115) exceeds the country-level regional total of 113 (Section 3.2.1) by two athletes; this is most plausibly explained by the same two Uzbek cyclists being entered in both Para Cycling Road (2 athletes) and Para Cycling Track (2 athletes), which would be double-counted in a discipline-level tally but counted once in the country-level roster. This reading is consistent with the IPC HIRA cycling entry tables for Paris 2024, which list two Uzbek competitors across Para Cycling as a whole rather than four separate individuals. This is the most likely explanation, though athlete-level IPC entry data were not available to verify it directly. Whether this concentration reflects a resource constraint or a deliberate strategic choice to concentrate limited resources in a small number of disciplines cannot be determined from participation counts alone; Section 4.4 interprets the pattern against the international literature, the SPLISS facilities pillar, and a regional policy comparator. No Central Asian country has appeared in wheelchair basketball, wheelchair rugby, or wheelchair tennis. Uzbekistan competed in cycling (road and track) for the first time at Paris 2024—a small but real sign of gradual diversification beyond the core disciplines; road and track are both governed by the UCI within the broader Para-cycling programme, of which hand cycling (for wheelchair-classified athletes) is a further, still-unrepresented discipline in the region.

**Figure 6:**
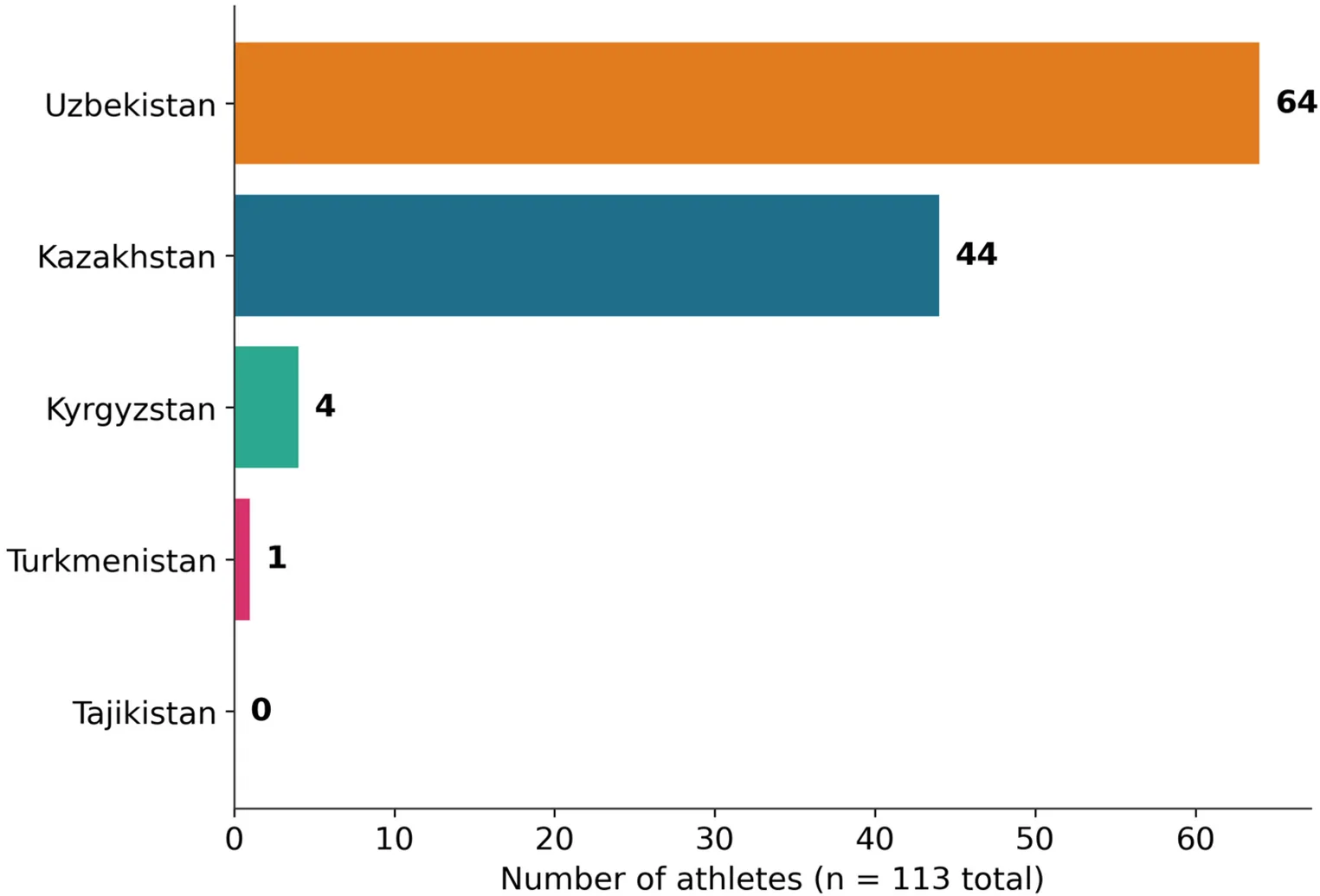
Central Asian Para athletes at Paris 2024, by country (*n* = 113; see Section 3.2.1). Source: IPC Historical Results Archive.

**Figure 7:**
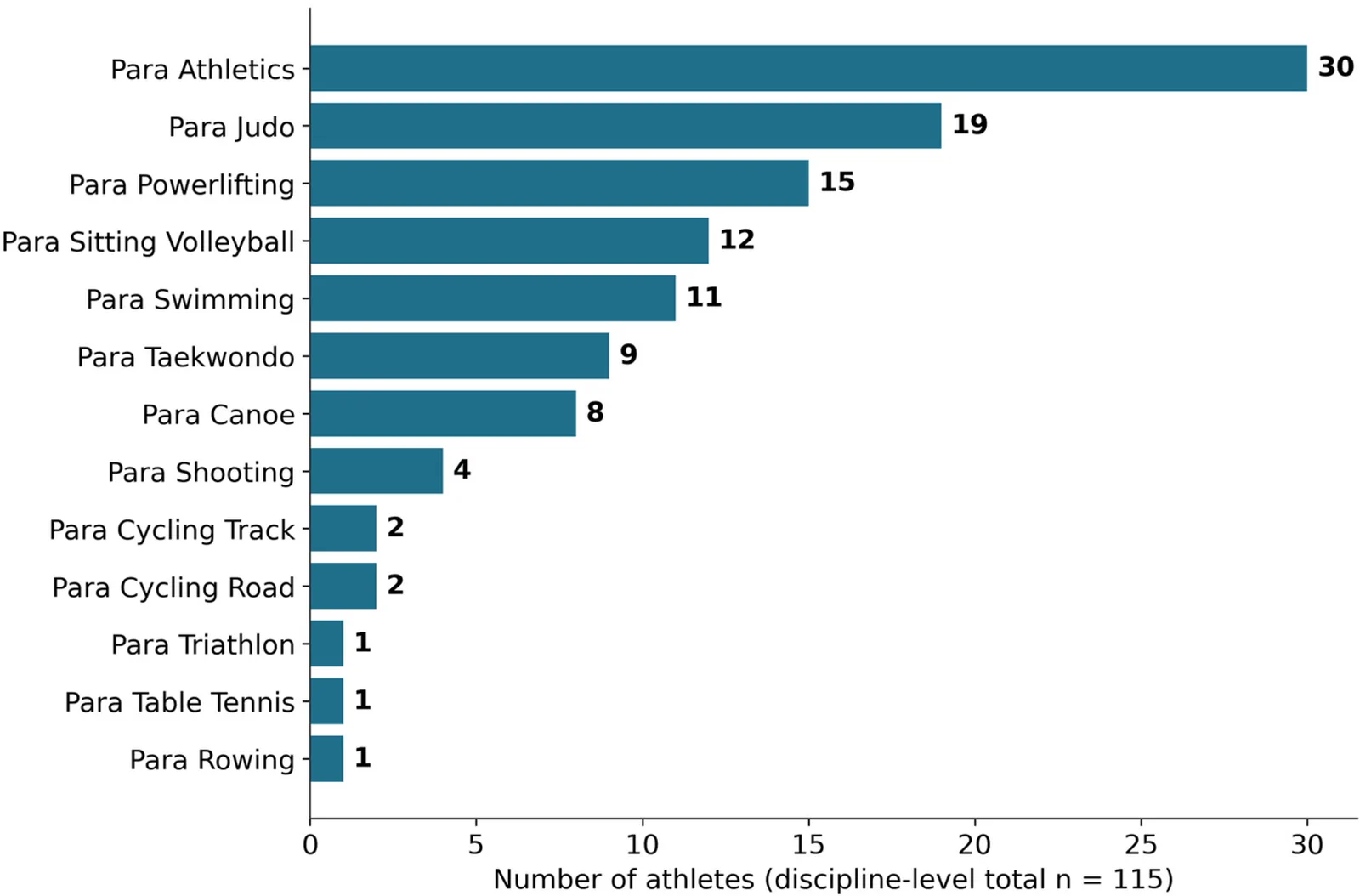
Central Asian Para athletes at Paris 2024, by discipline, based on IPC HIRA per-discipline participation tables for all 23 disciplines contested (discipline-level total *n* = 115, versus the country-level regional total of *n* = 113; see note in Section 3.2.3). Source: IPC Historical Results Archive (https://www.ipc-services.org/hira/paralympics/participants/code/PG2024).

#### 3.2.4 Medal efficiency

Table 1 reports athlete counts, female share, medal counts, and medal efficiency for all eight Summer Games editions.

Medal efficiency was 0% before 2012, rose to 4.2% (approximate 95% CI 0.7%–20.2%) at London 2012, then spiked sharply to 73.5% (approximate 95% CI 59.7%–83.8%) at Rio 2016—driven mainly by Uzbekistan, which won 34 medals from 32 athletes that edition, i.e., more medals than athletes—before falling back to 32.9% (23.2%–44.3%) at Tokyo 2020 and 31.0% (23.2%–40.0%) at Paris 2024 (Figure 3); as noted in Section 2.7, these intervals are heuristic uncertainty bands rather than formally correct binomial confidence intervals, since a single athlete can win more than one medal. The Rio 2016 interval does not overlap with London 2012, Tokyo 2020, or Paris 2024, indicating a genuine, edition-specific surge in medal-winning rather than sampling noise alone, though its cause is not established by these data; Tokyo 2020 and Paris 2024, by contrast, are statistically indistinguishable from each other. A logistic regression trend test across all eight editions (Section 2.7) shows an apparent overall increase in medal efficiency over time (*b* = 0.29 per edition, nominal *p* < 0.001), but this model shows severe overdispersion relative to a binomial process (Pearson *χ*^2^/df = 11.3), which is expected given the multiple-medals-per-athlete caveat noted above and confirms quantitatively that this is not a formally valid significance test; we report it only as a descriptive trend indicator alongside the edition-by-edition CIs. All medals came from Kazakhstan and Uzbekistan, in Para Athletics, Judo, Swimming, Taekwondo, Powerlifting, and Shooting.

#### 3.2.5 Summer versus Winter Games

Summer and Winter participation differ sharply (Figures 8, 9). Summer Games reached 113 athletes by 2024; Winter Games peaked at 9 at Milano Cortina 2026 (Table 3). Kazakhstan is the only country with continuous Winter participation since 1994; the 2018–2022 decline (8 to 5 athletes) and the subsequent rise to 9 in 2026 are discussed as a possible logistics/host-proximity effect in Section 4.2.

**Figure 8:**
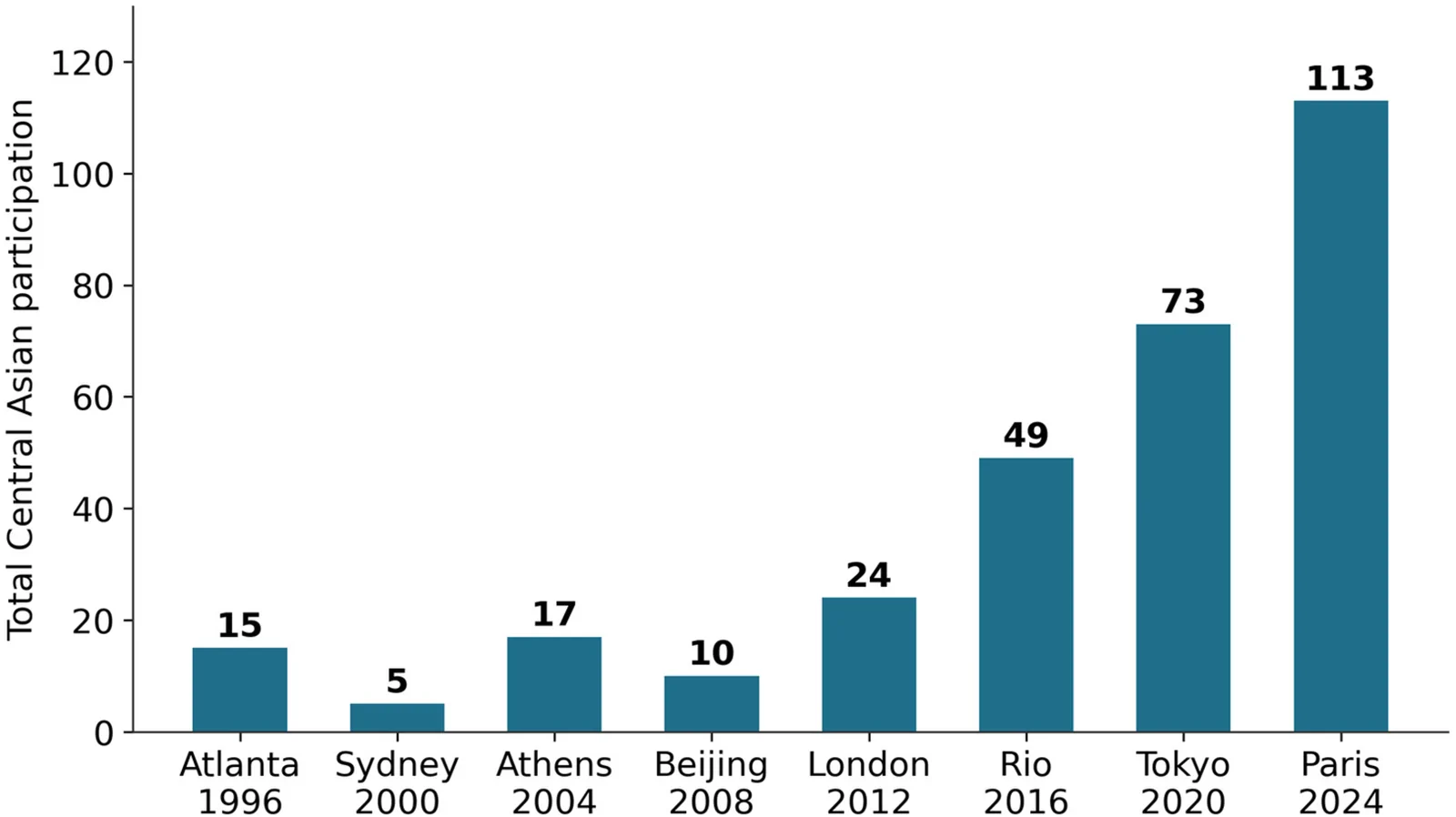
Central Asian participation in Summer Paralympic Games, 1996–2024. Source: IPC Historical Results Archive.

**Figure 9:**
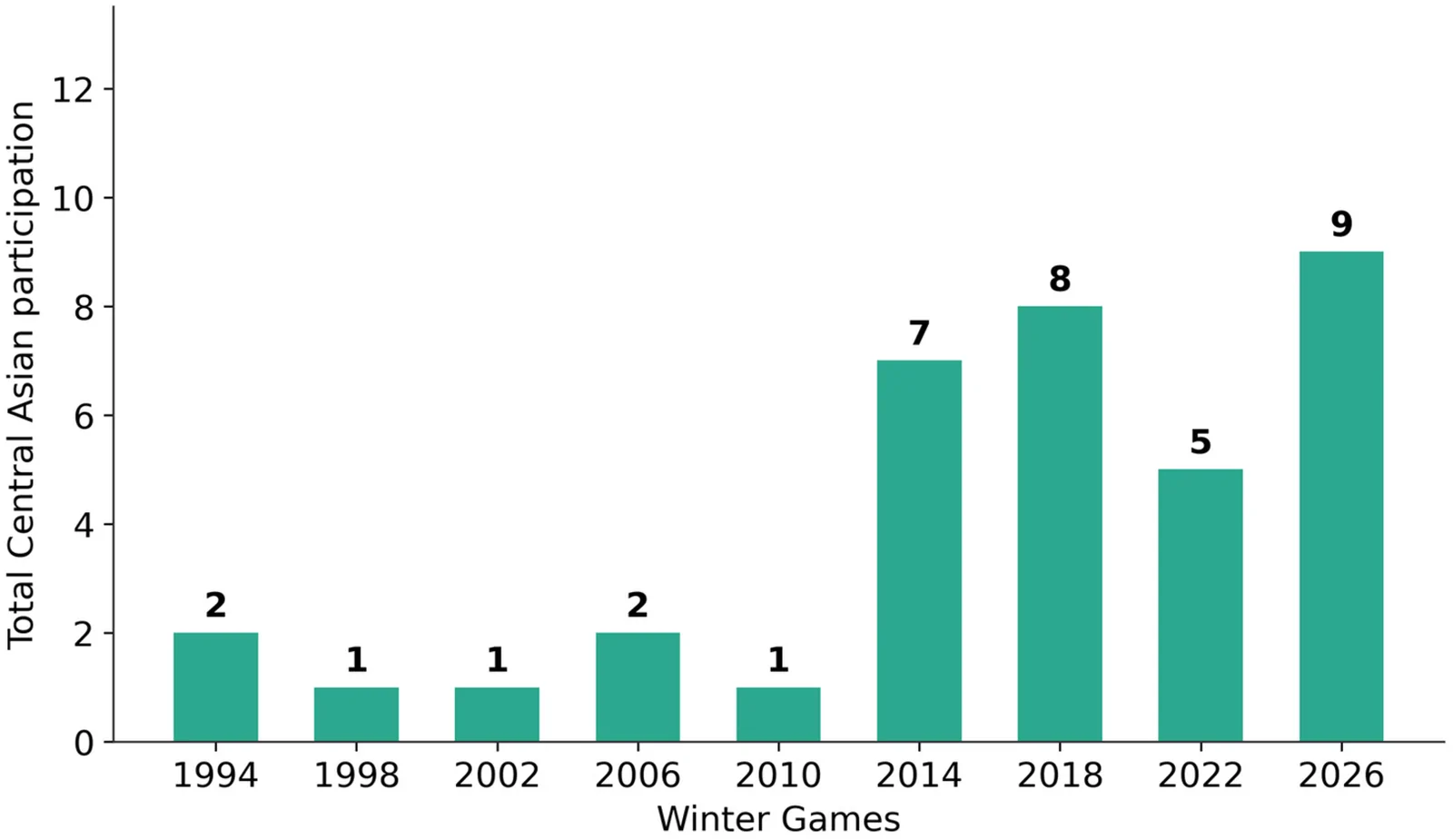
Central Asian participation in Winter Paralympic Games, 1994–2026. Source: IPC Historical Results Archive.

**Table 3:** Central Asian participation in Winter Paralympic Games (1994–2026).

Games	KAZ	UZB	KGZ	TJK	TKM	Total
1994	2	—	—	—	—	2
1998	1	—	—	—	—	1
2002	1	—	—	—	—	1
2006	2	—	—	—	—	2
2010	1	—	—	—	—	1
2014	5	2	—	—	—	7
2018	6	1	—	1	—	8
2022	5	—	—	—	—	5
2026	7	2	—	—	—	9

—, non-participation. Source: IPC Historical Results Archive.

## 4 Discussion

### 4.1 Literature coverage across regions

No prior study has combined a scoping review of Paralympic research with IPC archival data for Central Asia. The review revealed that the published literature originates almost entirely from high-income countries; our search identified no empirical study in a Q1–Q2 journal addressing Paralympic sport in Central Asia. Post-Soviet institutional legacies, a medical model of disability, restricted civic space, and specific cultural attitudes (14) differ from those in the countries studied to date. Marchibayeva (15) showed that even Kazakhstan's legislative and educational infrastructure for adaptive physical culture falls short of stated policy goals.

### 4.2 Competing explanations for participation growth

Three alternative or contributing explanations compete with a straightforward “programme growth” reading of the participation trend in Table 1 and Figure 4, and are considered together here because each could account for part of the same acceleration.

First, Central Asia's growth might be read as the reactivation of Soviet-era disability-sport infrastructure, given that the USSR is sometimes remembered as a dominant sporting power. This reading sits uneasily with the historical record. The Soviet Union officially denied the existence of disabled citizens (“there are no invalids in the USSR”) and boycotted the inaugural 1980 Paralympic Games in Arnhem, competing at the Paralympics for the first time only in 1988, under glasnost—roughly three decades after the countries that dominate the current literature had begun building Paralympic systems (32). Rehabilitation-oriented sport for disabled war veterans existed within Soviet disability societies, and informal domestic competitions reportedly continued in some constituent republics through the late Soviet period; the Kyrgyz National Paralympic Committee, for instance, traces local competitive activity to before its formal 1994 founding, although this specific claim rests on a single non-academic source. This parallel, medically framed system was never integrated into the international Paralympic movement, however, and left no elite-sport infrastructure comparable to what Western nations built over the same decades. Central Asia's post-2012 growth is therefore better understood as new institution-building on a genuinely thin post-Soviet base—consistent with the region's framing elsewhere in this article as an emerging Paralympic system, not the revival of a dormant Soviet one.

Second, the 2008–2016 and 2016–2024 acceleration phases overlap two IPC classification reforms: the 2013–2015 revision that produced the IPC Athlete Classification Code 2015 (in force through Paris 2024), and the 2018 World Para Athletics classification review, which introduced new sport classes and returned several existing classes to review status (Table 2). Reclassification can mechanically increase the number of athletes eligible to compete in a given class without reflecting a genuine expansion of the underlying athlete pool, and the overlap in timing means this cannot be ruled out as a partial contributor to the observed growth (33, 34).

Third, Paris 2024 was substantially closer, logistically and financially, to Central Asia than Rio de Janeiro 2016 or Tokyo 2020; part of the 2020–2024 increase (73 to 113 athletes) may reflect easier and cheaper qualification travel rather than underlying programme growth, a pattern also consistent with the Winter Games volatility in Table 3, where the largest Central Asian Winter delegation (2026) followed several editions of decline. This explanation is not fully consistent with the historical record, however: Beijing 2008 was the only other edition in the study period hosted in Asia, and if geographic proximity reliably increased Central Asian participation, that edition should also show a bump relative to neighboring editions. Instead, participation fell to 10 athletes at Beijing 2008—the second-lowest total in the series, down from 17 at Athens 2004—which runs counter to a simple distance-based account. Host proximity may still have contributed to the specific 2020–2024 increase alongside classification reform and the state-level measures discussed in Section 4.3, but it is not borne out elsewhere in the dataset, and is best read as one contributing factor among several.

### 4.3 Uzbekistan's acceleration: an institutional account

None of the three competing explanations in Section 4.2 fully accounts for Uzbekistan specifically. Uzbekistan's growth from zero athletes at Atlanta 1996 to 64 at Paris 2024—and specifically the +100% increase from 32 (Rio 2016) to 64 (Paris 2024)—coincides closely with a sequence of state interventions rather than a single policy shift. Presidential Decrees UP-5924 (24 January 2020) and UP-6099 (30 October 2020) established the general policy basis for expanding physical culture and sport, including for people with disabilities (38, 39). Presidential Resolution PP-5114 (18 May 2021) converted the National Paralympic Association into a National Paralympic Committee, created regional NPC branches, a dedicated Paralympic Faculty at the Uzbek State University of Physical Culture and Sport, a specialist training institute, and Paralympic sport centers in five regions (Karakalpakstan, Kashkadarya, Samarkand, Fergana, Tashkent) (40). The same resolution mandates, from 1 January 2022, additional quotas of up to 5% for athletes with disabilities referred to Olympic-reserve sports college departments (Article 10b), and stipulates that base tariff rates for coaches in Paralympic disciplines at state sports institutions be applied at one-and-a-half times the standard rate (Article 10b). PP-5114 additionally mandates, starting in 2022, the allocation of no less than 30 billion UZS annually in republican, regional, and district sport-event calendars for the preparation and conduct of Paralympic sporting events (Article 17). A further resolution, PP-389 (8 November 2024), extended free access to state sports facilities for people with disabilities through a dedicated social-service referral system (41). These measures directly preceded and accompanied the 2020–2024 growth phase (44 to 64 Uzbek athletes) and offer a plausible institutional mechanism for the acceleration—alongside, not instead of, the classification-reform and host-proximity effects discussed in Section 4.2. A direct comparison with Kazakhstan complicates a purely Uzbekistan-specific policy account, however: over the same 2016–2024 window, Kazakhstan's delegation grew from 11 to 44 athletes, a +300% increase—proportionally three times larger than Uzbekistan's +100% increase (32 to 64) over the identical period, despite no comparable, documented legislative package identified for Kazakhstan in this review. Read alongside each other, these two trajectories suggest that shared regional or global factors (classification reform, host proximity, the general expansion of the Paralympic programme documented in Section 1) plausibly account for a substantial share of both countries’ growth, and that Uzbekistan's documented state measures are better understood as adding to this shared regional trend—helping explain the scale of Uzbekistan's delegation in absolute terms—rather than as the sole or primary driver of its growth rate. This remains a plausible institutional account rather than a causal test: no independent evaluation data (athlete retention rates, budget execution, dropout) are publicly available to confirm that these specific measures, rather than other concurrent factors, drove the growth, and no equivalent legislative search was conducted for Kazakhstan, Kyrgyzstan, Tajikistan, or Turkmenistan, so we cannot rule out that comparable measures exist there and were simply not identified. Testing the mechanism would require primary-source policy analysis conducted in partnership with researchers from all five countries (Section 4.7).

### 4.4 Income-technology barrier and discipline concentration

The IPC data illustrate the inequalities in Oggero et al. (16). Although participation rose from 15 to 113 athletes, 56% of the regional delegation is concentrated in three disciplines—Para Athletics, Para Judo, and Para Powerlifting—with sitting volleyball, swimming, taekwondo, and canoe accounting for most of the remainder (Section 3.2.3); this limits both event range and medal ceiling. The presence of a full sitting-volleyball squad—a team sport requiring specialized wheelchairs, dedicated coaching and a twelve-athlete roster—alongside Para Canoe and Para Shooting complicates a purely equipment-cost account of concentration, since team sports carry coaching, travel, and roster-depth demands distinct from individual low-equipment disciplines; the pattern more plausibly reflects a mix of historical program investment and equipment access rather than equipment cost alone. Within the upper-middle income group, discipline concentration reflects equipment and infrastructure shortfalls not fully captured at the country-income level. Through the SPLISS lens, the facilities-and-equipment pillar remains the weakest for Central Asian countries; strengthening it would open further access to wheelchair sports and cycling, where Uzbekistan's first cycling road and track appearances at Paris 2024 offer an early signal of gradual diversification. Revuelta and Hernández (18) reports a structurally similar pattern in Latin America, where formal recognition of disability sport rights has outpaced investment in talent-identification and progression pathways, and Nagata et al. (42) find the same disconnect among ASEAN sport administrators in Southeast Asia, where elite Paralympic success is valued as a policy objective largely for its capacity to build public support rather than being backed by developed talent-pathway infrastructure—suggesting that discipline concentration in Central Asia may reflect a broader, income-transition-stage pattern shared across several regions rather than a feature unique to the region, though a direct medal-per-athlete comparison across regions was beyond the scope of the archival data assembled here.

### 4.5 Age profiles and talent identification

The data of Kons et al. (7) have practical implications. Countries building a Paralympic program could target disciplines with later peak ages—Para Powerlifting (Mdn = 36) and Para Archery (37)—where the wider age window gives more time for system development (43–47). Para Athletics (Mdn = 29), the primary Central Asian discipline, calls for earlier identification. The constraint-led model (5) predicts that limited athlete pools and classification capacity narrow entry points, making age-appropriate discipline selection one actionable strategy.

### 4.6 Policy transfer and adaptation

The ten interventions of Pankowiak et al. (12) cannot be applied without changes (48). In Central Asian countries, adaptation must address: the absence of developed parasport club systems; the implementation gap between policy and practice (49); the lack of trained classifiers; and limited disability knowledge in coach education. Marchibayeva (15) confirmed that workforce preparation and research output within Kazakhstan's existing legislation remain below the level needed—consistent with the coaching and research pillars of SPLISS.

This review draws primarily on five of SPLISS's nine pillars—participation, talent identification, coaching, facilities, and research—for which archival or published evidence was available. The competition-opportunities pillar can be partially assessed for Kazakhstan specifically: the country hosted the 2018 Para Judo World Cup (Atyrau) and the 2019 World Para Powerlifting Championships (Nur-Sultan) (50), a qualification event for Tokyo 2020, indicating some capacity to host, rather than only send athletes to, top-tier international competition in the region's two highest-medal-density disciplines. We were unable to establish an equivalent record for Uzbekistan, Kyrgyzstan, Tajikistan, or Turkmenistan, so this remains a partial, single-country data point rather than a regional assessment. Governance, financial support depth, and organizational structure are not systematically assessed here and are flagged as an explicit scope limitation (Section 4.8). Similarly, the constraint-led approach (5) is applied at the level of interpretation rather than measurement: impairment-type distribution, classification capacity (number of accredited classifiers per country), coaching-to-athlete ratios, and athlete-pool size upstream of Games qualification are not directly quantified in the archival IPC data used here, and the discipline-concentration finding in Section 3.2.3 should be read as consistent with, rather than proof of, constraint-led predictions. Revuelta and Hernández's (18) Latin American comparison reinforces this reading: countries in that region show the same pattern of stronger formal/normative recognition than operational talent-pathway infrastructure, which is closer to a structural-constraint account than to a purely strategic-choice account of discipline concentration, though the Central Asian data alone cannot resolve which explanation dominates.

### 4.7 Future directions

Four priorities emerge from the data. (1) Epidemiological studies documenting athlete numbers, impairment profiles, and barriers across all five countries; realistically a 2–3 year effort requiring cooperation from national Paralympic committees and ministries of health, and only feasible with data-sharing agreements that do not currently exist. (2) Policy analysis applying modified SPLISS and Paralympic models to the post-Soviet context, with stakeholder interviews conducted jointly with Central Asian researchers and institutions from the outset—not as translators or local facilitators for externally designed studies—to avoid the extractive dynamic that has been criticized elsewhere in international disability research. (3) Longitudinal development research benchmarked against the age patterns of Kons et al. (7) and the constraint-led model, tracking cohorts across at least two Games cycles (8 years) to distinguish genuine development from reclassification or host-proximity effects of the kind discussed in Section 4.2. (4) Culturally adapted psychological support programs addressing the motivation-hardiness findings of Krasmik et al. (17), piloted with national federations that currently lack permanent sport psychologists (51, 52). Additionally, including athlete qualification data and grey-literature sources—including the primary legislative sources identified in Section 4.3 for Uzbekistan, and equivalent documents for Kazakhstan, Kyrgyzstan, Tajikistan, and Turkmenistan where they exist—would strengthen future analyses; the near-absence of such sources for the latter three countries in the present review is itself a documented evidence gap (Section 4.8), not merely a search-strategy limitation.

These priorities should also be weighed against a broader question this review does not resolve: whether state investment in elite Paralympic sport is an unqualified good, or one claim among several on limited disability-related budgets that also include employment programs, healthcare, accessible transport, and the built-environment accessibility documented as lacking by Marchibayeva (15) and the UNDP (49) report on Central Asia. Elite Paralympic success is visible and diplomatically valuable (10), which may direct resources toward a small number of Games-eligible athletes ahead of broader disability-inclusion goals that benefit a much larger population. This review cannot adjudicate that trade-off with the data available, but future policy analysis conducted in the region (priority 2, above) should treat elite-sport investment as one competing claim on disability policy rather than as a self-evidently correct priority.

### 4.8 Limitations

This review was conducted by a single reviewer. Screening, full-text eligibility assessment, data charting and quality appraisal were carried out by the author alone, without independent duplicate screening or a formal consensus procedure, and single-reviewer selection carries a greater risk of eligible studies being missed than duplicate screening would. Identification combined a pre-existing project archive with database searching and AI-assisted retrieval, so repeating the search would not necessarily return an identical set of records. These constraints should be weighed when interpreting the size and composition of the included evidence base (*n* = 11); in particular, the absence of Q1–Q2 empirical work on Paralympic sport in Central Asia is the result of a single-reviewer search rather than an exhaustive determination. The records themselves are fully documented: every included study was read in full by the author, the complete charting table is provided as Supplementary Table S1, and every record identified at any stage is listed, with its eligibility status, in Supplementary Material S2.

This study is limited to English and Russian sources, which may have excluded local-language work; a systematic search of Kazakh, Uzbek, Kyrgyz, Tajik, and Turkmen-language sources returned almost no additional peer-reviewed material. This search also specifically targeted Russian-language academic repositories, including CyberLeninka; several potentially relevant records were located there, but the platform's access-verification step prevented retrieval of full text, and the remaining accessible Russian-language material on Paralympic sport in the region was institutional or journalistic rather than peer-reviewed. The near-absence of accessible peer-reviewed publishing on Paralympic sport in the regional languages is itself a finding of this review. The IPC analysis is descriptive and does not support causal claims; the confidence intervals reported in Section 3.2 quantify sampling uncertainty in small annual cohorts, not the effect of any specific policy or event. Athlete qualification data were unavailable, preventing impairment-level analysis. Discipline data for earlier Games are approximate, and the composition of the residual “Other” discipline category (Figure 7) could not be broken out at athlete-level resolution. HIRA data before 2014 are noted by the IPC itself as still under institutional review, which particularly affects the smallest early delegations (Atlanta 1996 to Beijing 2008) in Table 1. IPC records classify athletes by competition nation, not country of origin; Central Asian-born athletes who have relocated and compete for other national teams—a documented pattern in post-Soviet elite sport more broadly (53)—are not captured here and could lead this analysis to understate the region's true athletic talent pool. No specific cases were identified among Central Asian Para athletes; the point therefore concerns the limits of IPC nation-of-competition data. We were also unable to locate a comparable Eastern European regional analysis of Paralympic discipline concentration or national policy for the cross-regional comparison in Section 4.4; that comparison is therefore limited to Latin America (18) and Southeast Asia (42). Grey literature was only partially accessible. Stakeholder consultation—the optional sixth stage of Arksey and O’Malley—was not carried out.

## 5 Conclusion

This study combined a scoping review of Paralympic research with IPC participation data for Central Asia, focused on elite, Games-level sport. Participation grew from 15 to 113 athletes between 1996 and 2024, while medal efficiency followed a volatile rather than stable path—0% before 2012, a sharp spike to 73.5% at Rio 2016, and 31.0% (approximate 95% CI 23.2%–40.0%) at Paris 2024—so the evidence does not support a claim of either performance stabilization or dilution. A narrow discipline range, female representation of 41.6% (overlapping the approximate global benchmark of ∼43%; see Table 1), and minimal Winter Games involvement point to structural constraints that this review can describe but, with eight to nine data points per series, cannot fully explain. Viewed through the SPLISS pillars examined here and the constraint-led model, the data indicate shortfalls in equipment access, classification capacity, coaching, and psychological support, while Uzbekistan's documented 2019–2024 policy sequence offers one plausible, though unverified, institutional account of the region's most striking growth trajectory. The four priorities outlined here—pursued in partnership with, not merely about, Central Asian researchers and institutions—can guide evidence-based Paralympic development in the region.

## References

[1] Arksey H O'Malley L. Scoping studies: towards a methodological framework. Int J Soc Res Methodol. (2005) 8:19–32. 10.1080/1364557032000119616

[2] Levac D Colquhoun H O'Brien KK. Scoping studies: advancing the methodology. Implement Sci. (2010) 5:69. 10.1186/1748-5908-5-69 20854677 PMC2954944

[3] Tricco AC Lillie E Zarin W O'Brien KK Colquhoun H Levac D. PRISMA extension for scoping reviews (PRISMA-ScR): checklist and explanation. Ann Intern Med. (2018) 169:467–73. 10.7326/M18-0850 30178033

[4] De Bosscher V Shibli S Westerbeek H Van Bottenburg M. Successful Elite Sport Policies (SPLISS 2.0). Maidenhead: Meyer and Meyer (2015).

[5] Dehghansai N Lemez S Wattie N Pinder RA Baker J. Understanding the development of elite parasport athletes using a constraint-led approach. Front Psychol. (2020) 11:502981. 10.3389/fpsyg.2020.502981 33101110 PMC7554586

[6] Dehghansai N Lemez S Wattie N Baker J. A systematic review of influences on development of athletes with disabilities. Adapt Phys Act Q. (2017) 34:72–90. 10.1123/APAQ.2016-0030 28218871

[7] Kons RL Apollaro G Bevins R Morales J Gulias VO Grosprêtre S. Age distributions in Paralympic Games Paris 2024. Front Sports Act Living. (2026) 7:1720957. 10.3389/fspor.2025.1720957 41660058 PMC12872764

[8] Fadian UFL Hidayatullah MF Riyadi S Umar F. Development of Paralympic sport 2008-2022 through systematic literature review. KnE Soc Sci. (2024) 9:533–44. 10.18502/kss.v9i19.16545

[9] United Nations. Convention on the Rights of Persons with Disabilities. New York, NY: United Nations (2006).

[10] Beacom A Brittain I. Public diplomacy and the International Paralympic Committee. Diplomacy Statecraft. (2016) 27:273–94. 10.1080/09592296.2016.1169795

[11] Patatas JM De Bosscher V De Rycke J Winckler C Legg D. Elite disability sport policies: a comparison. Int J Sport Policy Polit. (2018) 10:655–72.

[12] Pankowiak A Brockett C De Bosscher V Westerbeek H. National Paralympic sport policies. Int J Sport Policy Polit. (2023) 15:435–55. 10.1080/19406940.2023.2196992

[13] McBean C Schofield G Schluter P Watson P. Disability sport policy in New Zealand. Int J Sport Policy Polit. (2022) 14:207–25.

[14] Katsui H. Towards Equality: Creation of the Disability Movement in Central Asia. Helsinki: Helsinki University Press (2005).

[15] Marchibayeva US. Development of adaptive physical culture and sports in the educational system of the republic of Kazakhstan. Bull Natl Acad Sci Repub Kazakhstan. (2024) 5:148–64. 10.32014/2024.2518-1467.832

[16] Oggero G Puli L Smith EM Khasnabis C. Participation and achievement in the Summer Paralympic Games. Sustainability. (2021) 13:11758. 10.3390/su132111758

[17] Krasmik Y Aimaganbetova O Iancheva T. Motivation and hardiness in Paralympic sport. KazNU Bull Psychol Sociol Ser. (2024) 3:38–50. 10.26577/JPsS.2024.v90.i3.04

[18] Revuelta B Hernández R. Public policies for the promotion of Paralympic sport in Latin America: a comparative analysis of 10 countries. Front Sports Act Living. (2026) 8:1844536. 10.3389/fspor.2026.1844536 42338997 PMC13284080

[19] Munn Z Peters MDJ Stern C Tufanaru C McArthur A Aromataris E. Systematic review or scoping review? Guidance for authors when choosing between a systematic or scoping review approach. BMC Med Res Methodol. (2018) 18:143. 10.1186/s12874-018-0611-x 30453902 PMC6245623

[20] National Paralympic Committee of the Republic of Kazakhstan. Official website (2024). Available online at: https://paralympic.kz/ (Accessed January 20, 2026).

[21] National Paralympic Committee of Uzbekistan. Official website (2024). Available online at: http://paralympic.uz/ (Accessed January 20, 2026).

[22] Patatas JM De Rycke J De Bosscher V Kons RL. It’s a long way to the top. Adapt Phys Act Q. (2021) 38:605–25. 10.1123/apaq.2020-0147 34404027

[23] Thomas N Guett M. Disability sport policy. In: Henry I Ko L-M, editors. Routledge Handbook of Sport Policy. London: Routledge (2014). p. 295–307.

[24] Hammond AM Jeanes R. Federal government involvement in Australian disability sport, 1981–2015. Int J Sport Policy Polit. (2018) 10:189–207. 10.1080/09523367.2017.1337000

[25] Hong QN Pluye P Fàbregues S Bartlett G Boardman F Cargo M. Mixed Methods Appraisal Tool (MMAT), Version 2018. Registration of Copyright (#1148552). Ottawa: Canadian Intellectual Property Office, Industry Canada (2018).

[26] International Paralympic Committee. IPC Historical Results Archive (HIRA): country and Games results, 1960–present (2026). Available online at: https://www.ipc-services.org/hira/ (Accessed April 15, 2026).

[27] Cohen JW. Statistical Power Analysis for the Behavioral Sciences. 2nd ed. Hillsdale, NJ: Lawrence Erlbaum (1988).

[28] Dehghansai N Pinder RA Baker J. Talent identification and development in Paralympic contexts. Front Sports Act Living. (2022) 4:926974. 10.3389/fspor.2022.926974 35784802 PMC9247143

[29] Dehghansai N Pinder RA Baker J. Looking for a golden needle in the haystack. Front Sports Act Living. (2021) 3:635977. 10.3389/fspor.2021.635977 33898986 PMC8060486

[30] Houlihan B Chapman P. Talent identification and development in elite youth disability sport. Sport Soc. (2016) 19:1237–52. 10.4324/9781351271882-7

[31] Santos LG Ramos BL Ferreira VTK Cidade RE Winckler C. Towards equity? The trajectory of women’s participation in the Summer Paralympic Games (1988–2024). Front Sports Act Living. (2025) 7:1593956. 10.3389/fspor.2025.1593956 40661674 PMC12256459

[32] Phillips SD. “There are no invalids in the USSR!” A missing Soviet chapter in the new disability history. Disabil Stud Q. (2009) 29(3):936. 10.18061/dsq.v29i3.936

[33] Mann DL Tweedy SM Jackson RC Vanlandewijck YC. Classifying the evidence for evidence-based classification. J Sports Sci. (2021) 39:1–6. 10.1080/02640414.2021.1955523 34369294

[34] Kons RL Patatas JM. Paralympic sports classification: need for research. Am J Phys Med Rehabil. (2023) 102:929–30. 10.1097/PHM.0000000000002264 37104644

[35] International Paralympic Committee. IPC Athlete Classification Code and International Standards. Bonn: IPC (2015).

[36] International Paralympic Committee. World Para Athletics Classification Rules and Regulations. Bonn: IPC (2018). Available online at: https://www.paralympic.org/athletics/classification (Accessed January 20, 2026).

[37] International Paralympic Committee. IPC Athlete Classification Code, Version 1 January 2025. Bonn: IPC (2025). Available online at: https://www.paralympic.org/sites/default/files/2025-02/IPC%20Classification%20Code%2001_01_2025.pdf (Accessed January 20, 2026).

[38] Republic of Uzbekistan. Decree of the President No. UP-5924, 24 January 2020, “On measures for further improvement and popularization of physical culture and sport in the Republic of Uzbekistan.” Tashkent (2020). Available online at: https://lex.uz/ru/docs/4711329 (Accessed January 20, 2026).

[39] Republic of Uzbekistan. Decree of the President No. UP-6099, 30 October 2020, “On measures to introduce a healthy lifestyle.” Tashkent (2020). Available online at: https://lex.uz/ru/docs/5077669 (Accessed January 20, 2026).

[40] Republic of Uzbekistan. Resolution of the President No. PP-5114, 18 May 2021, “On additional measures for the development of the Paralympic movement.” Tashkent (2021). Available online at: https://lex.uz/ru/docs/5426281 (Accessed January 20, 2026).

[41] Republic of Uzbekistan. Resolution of the President No. PP-389, 8 November 2024, “On additional measures for enhancement of the system of physical rehabilitation through involvement in sport of persons with disability.” Tashkent (2024). Available online at: https://lex.uz/ru/docs/7215999 (Accessed January 20, 2026).

[42] Nagata S Bloyce D Sato T Okade Y. It’s about inspiring the greater community to continue supporting this sector: elite sport success as a main policy objective for disability sport promotion in ASEAN member states. Int J Sport Policy Polit. (2023) 15:655–70. 10.1080/19406940.2023.2236635

[43] Schipman J Gallo P Marc A Antero J Toussaint J-F Sedeaud A. Age-related changes in Para and wheelchair racing. Front Physiol. (2019) 10:256. 10.3389/fphys.2019.00256 30949064 PMC6436002

[44] Hogarth L Nicholson V Payton C Burkett B. Modelling the age-related trajectory of performance in para swimmers. Scand J Med Sci Sports. (2021) 31:925–35. 10.1111/sms.13910 33345411

[45] Severin AC Baumgart JK Haugen T Hogarth L. Peak age and performance trajectories in Para powerlifters. Am J Phys Med Rehabil. (2023) 102:645–52. 10.1097/PHM.0000000000002051 35687764

[46] Costa RRG Neto FR Ramos BL Tanhoffer R O'Shea B Winckler C. Age-related trajectory in wheelchair rugby. Am J Phys Med Rehabil. (2024) 103:747–52. 10.1097/PHM.0000000000002434 38206584

[47] Ribeiro Neto F Teixeira Fabrício dos Santos LG Castelli Correia de Campos LF Winckler C Gomes Costa RR. Paralympic versus Olympic canoe sprint: comprehension of the development trajectory of the kayak 200 meters. Percept Mot Skills. (2024) 131:1291–307. 10.1177/00315125241247859 38669451

[48] Dowling M Brown P Legg D Grix J. Living with imperfect comparisons. Sport Manag Rev. (2017) 21:101–13. 10.1016/j.smr.2017.05.002

[49] Schulze M Kabir AHM. Promoting the Rights of Persons with Disabilities in Central Asia: Institutional Experiences and the Way Forward. Lessons Learned Series: Rule of Law, Justice and Human Rights. Bratislava: United Nations Development Programme Regional Centre for Europe and the CIS (2013).

[50] International Paralympic Committee. Nur-Sultan 2019 (formerly Astana 2019) – World Para Powerlifting Championships (2019). Available online at: https://www.paralympic.org/sites/default/files/document/190726081511853_Nur-Sultan+2019+WCH+results+book.pdf (Accessed September 12, 2026).

[51] Fletcher D Sarkar M. A grounded theory of psychological resilience in Olympic champions. Psychol Sport Exerc. (2012) 13:669–78. 10.1016/j.psychsport.2012.04.007

[52] Nascimento Junior JRA Freire GLM Granja CTL Barros NP Oliveira DV Trevisan LG. The role of resilience on motivation among Brazilian parathletes. J Phys Educ. (2020) 32:1–10. 10.4025/jphyseduc.v32i1.3201

[53] Skey M. Sport and nationality: towards thick and thin forms of citizenship. Natl Identities. (2020) 23:1–16. 10.1080/14608944.2020.1815421

