# Supplementary material for: Paralympic sport in Central Asia: a scoping review with descriptive analysis of participation, achievement, and medal efficiency (1996–2024)
Source: Front Sports Act Living. 2026 Sep 22;8:1893368. doi: 10.3389/fspor.2026.1893368 (PMC13640612; doi:10.3389/fspor.2026.1893368)
Supplement: Supplementary file 1 [file Table3.docx]

# PRISMA-ScR Checklist

*“Paralympic sport in Central Asia: a scoping review with descriptive analysis of participation, achievement, and medal efficiency at the elite (Games) level (1996-2024)”. Based on the PRISMA Extension for Scoping Reviews (PRISMA-ScR) checklist (Tricco et al., 2018).*

| **#** | **Section / Topic** | **Checklist item** | **Reported in manuscript** |
| --- | --- | --- | --- |
| 1 | TITLE | Identify the report as a scoping review. | Title includes “a scoping review with descriptive analysis”. |
| 2 | ABSTRACT / Structured summary | Provide a structured summary including background, objectives, eligibility criteria, sources of evidence, charting methods, results, and conclusions. | Abstract. |
| 3 | INTRODUCTION / Rationale | Describe the rationale in the context of what is already known; explain why the review questions/objectives lend themselves to a scoping review. | Section 1 (Introduction); Section 1.1 states why a scoping design and dual-source approach were chosen. |
| 4 | INTRODUCTION / Objectives | Provide explicit statement of the questions and objectives, articulating PCC (Population, Concept, Context). | Section 1.1 (aims); Section 2.2 (RQ1-RQ3). |
| 5 | METHODS / Protocol and registration | Indicate whether a review protocol exists, if/where it can be accessed, and if available, provide registration information. | No review protocol was registered prospectively; this is stated in Section 2.1. The review questions (Section 2.2), eligibility criteria (Section 2.4), selection process (Section 2.5) and charting process (Section 2.6) are reported in full, and the record-level source audit is provided as Supplementary Material S2. |
| 6 | METHODS / Eligibility criteria | Specify inclusion/exclusion criteria, giving rationale. | Section 2.4. |
| 7 | METHODS / Information sources | Describe all information sources, with dates of coverage. | Section 2.3 (Scopus, WoS, SPORTDiscus, Jan 2010-Mar 2026; citation chasing; project bibliography; grey literature; NPC websites). |
| 8 | METHODS / Search | Present full search strategy for at least one database, including limits. | Section 2.3 (Block 1 / Block 2 Boolean structure); full per-database syntax not reproduced verbatim in-text — available on request / recommended as a supplementary appendix if the author retains the original query strings. |
| 9 | METHODS / Selection of sources of evidence | State the process for selecting sources (screening, eligibility, inclusion in the review). | Section 2.5 (Selection of sources of evidence) — new subsection. States that screening was conducted by the sole author (UM), that it was not performed independently in duplicate, that no between-reviewer discrepancies arose and no arbitration procedure was applicable, and that uncertain relevance was resolved in favour of retention for full assessment. Two identification routes reported separately. Figure 1; Supplementary Material S2. |
| 10 | METHODS / Data charting process | Describe methods of charting data, number of reviewers, and any processes for obtaining/confirming data. | Section 2.6 (Data charting process). Charting by the sole author (UM); not performed in duplicate and not independently verified; no reconciliation procedure required. Handling rule for ambiguous values stated (recorded as reported; left blank rather than inferred; no author contact). MMAT appraisal by the same single reviewer, non-exclusionary. Single-reviewer design acknowledged as a limitation in Section 4.8. |
| 11 | METHODS / Data items | List and define all variables charted. | Section 2.6 (author, year, journal, quartile, context, design, sample, domain, key findings, MMAT rating, Central Asian relevance) — see Supplementary Table S1 for the completed charting table. |
| 12 | METHODS / Critical appraisal of individual sources of evidence | If done, provide a description of the methods used and how this information was used. | Section 2.6 (MMAT, Hong et al. 2018), applied descriptively without exclusion; appraisal conducted by the sole author without duplicate appraisal. |
| 13 | METHODS / Synthesis of results | Describe the methods of handling and summarizing the data that was charted. | Section 3.1 (narrative synthesis by domain D1-D3 plus psychological/regional); Section 3.2 (quantitative descriptive analysis with Wilson CIs and trend tests, Section 2.6). |
| 14 | RESULTS / Selection of sources of evidence | Give numbers of sources screened, assessed for eligibility, and included, with reasons for exclusions, ideally with a flow diagram. | Figure 1 — two-route PRISMA-ScR flow diagram. Route 1: 102 records identified -> 38 duplicates removed -> 64 unique assessed in full text -> 58 excluded, 6 included. Route 2: 5 records identified via other methods -> 5 assessed -> 0 excluded, 5 included. Total charted n = 11. Reasons for exclusion given in Figure 1 and at record level in Supplementary Material S2. Also Section 2.5 and Section 3.1.1. |
| 15 | RESULTS / Characteristics of sources of evidence | For each source, present characteristics for which data were charted. | Supplementary Table S1. |
| 16 | RESULTS / Critical appraisal within sources of evidence | If done, present data on critical appraisal of included sources. | Supplementary Table S1, MMAT column (design-level appraisal; not exclusionary). |
| 17 | RESULTS / Results of individual sources of evidence | For each included source, present the relevant data charted that relate to the review questions/objectives. | Section 3.1.1-3.1.5 (narrative, by domain); Supplementary Table S1 (tabular). |
| 18 | RESULTS / Synthesis of results | Summarize and/or present the charting results as mapped to the review questions/objectives. | Section 3.1 (literature map) and Section 3.2 (IPC archival findings), read together per Section 2.1. |
| 19 | DISCUSSION / Summary of evidence | Summarize the main results, link to the review questions/objectives, and consider the relevance to key groups. | Section 5 (Conclusion); target audience stated in Section 1. |
| 20 | DISCUSSION / Limitations | Discuss the limitations of the scoping review process. | Section 4.8. Opens with a dedicated paragraph on the single-reviewer design: screening, charting and appraisal all by the sole author; no duplicate screening, duplicate charting or consensus procedure; no calibration or repeat screening; exclusion reasons not logged contemporaneously; AI assistance during identification and screening; implications for the interpretation of the evidence base (n = 11). |
| 21 | DISCUSSION / Conclusions | Provide a general interpretation of the results with respect to the review questions and objectives, as well as potential implications and/or next steps. | Section 4.7 (future directions) and Section 5 (Conclusion). |
| 22 | FUNDING | Describe sources of funding for the included sources of evidence, as well as sources of funding for the scoping review; describe the role of the funders. | Funding statement: no financial support received for this review. |
